# *Candida auris*: Epidemiology Update and a Review of Strategies to Prevent Spread

**DOI:** 10.3390/jcm13226675

**Published:** 2024-11-07

**Authors:** Justin F. Hayes

**Affiliations:** 1 Division of Infectious Diseases, Department of Medicine, College of Medicine, University of Arizona, Tucson, AZ 85724, USA; justinhayes@email.arizona.edu; Tel.: +1-520-626-6887; Fax: +1-520-626-5183; 2 Antimicrobial Stewardship Program, Banner University Medical Center-Tucson and South, 1501 N. Campbell Avenue, P.O. Box 245039, Tucson, AZ 85724, USA

**Keywords:** *Candida auris*, *C. auris*, candidemia, candidiasis, public health, epidemiology

## Abstract

Candida auris (*C. auris*) has emerged as a fungal pathogen with great propensity to spread rapidly on a global scale. *C. auris* infections have also caused significant morbidity and mortality. Strategies to prevent spread and outbreaks are critical. In this review, an update on the epidemiology of *C. auris* and a discussion of strategies to combat the spread of *C. auris* are presented. Future directions are also discussed.

## 1. Introduction

*Candida auris* (*C. auris*) first emerged in 2009 and was quickly recognized to possess the ability to spread rapidly and produce multidrug resistance [1]. *C. auris* has the notable ability to persist under the most challenging conditions, creating a setting difficult for its eradication [2]. In addition, *C. auris* has been associated with serious infections leading to increased mortality [3,4]. Due to these characteristics, *C. auris* has received global recognition from the World Health Organization as a critical fungal priority pathogen [5]. This status comes as the Centers for Disease Control and Prevention (CDC) reported 2377 clinical cases and 5754 screening cases of *C. auris* in the United States in 2022 [6].

In this review, *C. auris* epidemiology, propensity for outbreaks, and current prevention strategies are discussed in detail. Finally, future directions to address the continued threat of *C auris* globally are outlined. To perform the literature review, PubMed/Medline was reviewed for relevant articles related to *C. auris* from its inception to 1 October 2024. Increased attention was focused on relevant articles published within the last 5 years. In addition, information from relevant public health and global health agencies, such as the CDC, was reviewed for inclusion in the article.

## 2. *Candida auris* Biology, Commensalism, and Pathogenesis

*C. auris* is a haploid yeast that is phylogenetically related to *Candida haemulonii* and *Candida lusitaniae*. It can undergo dimorphism with an ability for filamentation and phenotypic switching [7,8,9]. More specifically, it can exist as either a unicellular, budding yeast or as multicellular aggregates [10]. Of critical importance, *C. auris* has been misidentified as *Candida haemulonii* or other *Candida* species by traditional phenotypic and biochemical methods. Fortunately, advancements in laboratory detection methods have improved our ability to make an accurate identification of *C. auris* [3,11].

*C. auris* has a near impeccable ability for asymptomatic colonization, with estimates showing that 90–95% of humans colonized with *C. auris* are asymptomatic [12]. *C. auris* encodes genes that produce adhesin families, with *C. auris*-specific adhesin SCF1 and conserved adhesin IFF4109 emerging as significant contributors to the long-term colonization and infection of both biological and abiotic surfaces [13]. An interesting feature of *C. auris* is the ability of the organism to have either a commensal or pathogenic lifestyle. Experts have stated that the adoption of either a pathway of commensalism or pathogenesis depends on the immune status of the host, the local tissue environment, the genetic potential of the organism, the organism’s epigenetic profile, and the microbial consortium at the site of adherence [14].

Most notably, *C. auris* has the propensity to colonize the skin and interact with other members of the skin microbiota. This has been an area of scientific interest. In one study, genomic, clinical, and epidemiologic data from nearly 4000 samples collected from 57 residents over a 3-month period were studied. The analysis demonstrated elevated *C. auris* bioburden on the nares, palms, and fingertips [15]. Further work examining dynamic interactions of *C. auris* with other skin microbiota and the ability to achieve both lifestyles of commensalism and pathogenesis will shed new light on *C. auris*. Proctor et al. have specifically discussed a damage response framework being applied to *C. auris*, as it highlights the ability of members of the microbiota to undergo the adoption of specific lifestyles, such as commensal or pathogenic lifestyles. The authors further note consensus gathering around the idea of *C. auris* undergoing micro-diversification on its host for months [14]. There still remain many unanswered questions regarding *C. auris*’s ability to achieve both commensalism and pathogenesis.

## 3. Epidemiology

Genomic analyses have demonstrated that *C. auris* emerged on several continents simultaneously with six distinct clades. Initially, four clades were described: Clade I (South Asian), Clade II (East Asian), Clade III (South African), and Clade IV (South American) [16]. More recently, two additional clades have been described: Clade V (Iran) and (Clade VI) (Singapore) [17,18,19]. The clades are genetically separated with even differences in strains within the same clade differing by thousands of single-nucleotide polymorphisms. Further, Clade IV has been noted to have the most significant virulence, while Clade II has demonstrated less pathogenicity in infection models compared to the other clades [10]. Over time, *C. auris* infections have been described in more than 40 countries, with a significant increase in the identification of initial cases in many countries during the Coronavirus Disease 2019 (COVID-19) pandemic [20]. Increased stress on health systems and the overcrowding of hospitals globally were likely contributors to the growth in cases during this period of time.

Clinically, *C. auris* presents, like other *Candida* spp., as a spectrum from mucosal infections to life-threatening invasive infections. These presentations consist not only of candidemia (i.e., bloodstream infection) but also invasive candidiasis (i.e., abdominal candidiasis, osteomyelitis, ocular candidiasis), leading to significant morbidity and mortality [1,21]. In one early report evaluating 54 patients with *C. auris* infection from multiple countries, diabetes mellitus was the most common comorbidity, with half of patients having surgery within the prior 90 days of positive culture for *C. auris*. In addition, 73% of patients had a central venous catheter in place and 61% had a urinary catheter in place [22]. *C. auris* has caused candidemia, wound infections, ear infections, and genitourinary infections, while also having an association with contamination of medical devices [23,24]. This is in line with other *Candida* spp. in that *C. auris* mainly causes healthcare-associated infections. However, in contrast to other *Candida* spp., *C. auris* epidemiology is notable for the discrepancy in the burden of disease with only isolated cases reported in certain regions and large outbreaks reported in other geographic locations [25].

In terms of mortality, *C. auris* candidemia crude in-hospital mortality has varied from 25% to 70% [20]. As a major contributing factor, several isolates of *C. auris* have been found to contain resistance to major classes of antifungal agents, including polyenes, azoles, and echinocandins [26]. This fact, along with the ability for *C. auris* to spread rapidly in healthcare settings, sets the stage for increased mortality risk. To further evaluate risk factors for poor outcomes in *C. auris* candidemia, Jimenez and colleagues performed a multicenter cohort study in the United States. In total, 187 patients from 5 health systems were included, and the results demonstrated an overall mortality of 54.6%. Survivors had lower Pitt bacteremia scores, less use of hemodialysis, less prior known colonization with *C. auris* or carbapenem-resistant pathogens, and less presence of indwelling devices. In addition, multivariable logistic regression analysis revealed Pitt bacteremia score and dialysis as predictors of mortality. The authors concluded that a set of both acute and chronic risk factors led to poor outcomes in *C. auris* infections [27].

Although the epidemiology of *C. auris* is not as well described as other *Candida* spp., new global updates have come into focus. In the United States, Lyman and colleagues described an increase in clinical cases of *C. auris* that occurred from 2019 to 2021. The investigators demonstrated a 44% increase in clinical cases in 2019 compared to previous years, which then increased by 59% in 2020 and an additional 95% in 2021. Additionally, the highest number of new states (eight) reported their first case in 2020 with an additional three states reporting their first case in 2021. A particularly worrisome part of the study was the identification of increasing echinocandin resistance, which is first-line therapy for *C. auris* infections [28]. In Europe, outbreaks have occurred in the United Kingdom, Spain, and Italy, with sporadic reports of cases from multiple other European countries [29]. Additionally, the European Confederation of Medical Mycology (ECMM) has collected epidemiologic data and clinical outcomes of patients with culture-proven candidemia in Europe as part of a collaborative candidemia study. In total, 64 hospitals from 20 countries participated and *C. auris* was responsible for 3% of the cases. In addition, *C. auris*, as the cause of candidemia, was found to predict mortality [30]. In another region of the world, a report from South Africa demonstrated that during 2012–2016, a dramatic increase in *C. auris* cases occurred. A total of 1692 confirmed or probable cases of *C. auris* were reported in both public and private hospitals, with most cases being admitted to private hospitals. Of cases with a recorded specimen type, 29% had invasive disease with isolates reported from blood, tissue, body fluid, and cerebrospinal fluid. The rest of the isolates were reported from sites of probable colonization, including the respiratory tract, urine, and skin. Of cases with a known location, 92% were reported from Gauteng Province, a densely populated location and travel hub [31]. In another study from South Africa, investigators determined the incidence risk for candidemia and the proportion of candidemia cases due to *C. auris* during 2016–2017. A total of 14% of candidemia cases were due to *C. auris*, while the incidence risk of all candidemia and *C. auris* fungemia was 83.8 (95% CI, 81.2–86.4) cases/100,000 admissions and 1.96 (95% CI, 1.4–2.6) cases/100,000 admissions, respectively. Prior systemic antifungal therapy was associated with 40% increased adjusted odds of *C. auris* fungemia, while the in-hospital case fatality ratio did not differ among species. The incidence of *C. auris* fungemia was again highest in Gauteng Province [32]. Finally, Prayag et al. shed light on the emergence of *C. auris* in India through a retrospective evaluation of 79 cases of candidemia. *C. auris* was found to be the most common species identified (43.03%), with all *C. auris* isolates demonstrating fluconazole resistance and 32.35% being resistant to amphotericin B. Crude mortality was higher for patients with *C. auris* fungemia compared to patients with bloodstream infections due to other *Candida* spp. [33].

## 4. Outbreak Capability

As the previous section indicates, *C. auris* can promote surges of new cases and outbreaks. In terms of its rapid emergence, mycology experts proposed a hypothesis stating that *C. auris* is an example of a new pathogenic fungus that emerged due to global warming [34]. In addition, *C. auris* possesses notable features, including thermal adaptation and saline tolerance, that allow for survival outside of a human host [20]. Since its emergence, challenges with outbreaks of *C. auris* have been described in several settings, including long-term and chronic care facilities, adult intensive care units (ICUs), neonatal ICUs, and correctional facilities [35,36,37,38].

Mechanisms for *C. auris*’ transmissibility have been described. In particular, the persistence of *C. auris* has been a significant concern in healthcare settings. One surveillance study described a weekly hospital screening program that demonstrated persistent colonization of patients with *C. auris*, with 34% (17/50) of patients in the cohort having at least one negative result followed by a positive result [39]. To explain this degree of persistence, antifungal-tolerant biofilm formation in *C. auris* has been elucidated as one potential mechanism [40]. For example, Sherry and colleagues studied the biofilm forming capacity of aggregative and non-aggregative *C. auris* phenotypes and characterized the antifungal susceptibility of the isolates. The investigations demonstrated the ability of *C. auris* to adhere to surfaces, form biofilms, and produce resistance to antifungals, including caspofungin, that were active against their planktonic counterparts [41]. In another study, investigators evaluated the biofilm matrix of a set of *C. auris* isolates. The analysis demonstrated that the biofilm of *C. auris* was rich in mannan-glucan polysaccharides like other *Candida* species, with a notable ability to sequester drugs. Additionally, there was variability in the content of the mannan and glucan polymers noted within the *C. auris* biofilm matrix during the experiments [42].

In addition to and related to the persistence of *C. auris*, environmental contamination is another area of concern. In one study focused on *C. auris*’ environmental contamination, investigators demonstrated that *C. auris* contamination is rapid after the disinfection of surfaces near *C. auris*-colonized patients. Further, having a higher number of *C. auris*-colonized body sites was associated with greater odds of environmental colonization [43]. This ability for rapid environmental contamination was postulated to come, in part, from *C. auris’s* skin tropism. To add to the evidence base for this mechanism, a murine translational model showed that *C. auris* had a striking ability to maintain long-term skin tissue compartment colonization and residence [44]. In theory, *C. auris* could exist at a deeper skin level rather than on the surface of the skin. This could explain why individuals colonized with *C. auris* test negative at one time point but positive at another time point.

## 5. Strategies to Prevent Spread

The collection of the characteristics and traits of *C. auris* provides a critical need for strict infection prevention protocols, as well as additional strategies to prevent spread. The rest of this section will review various strategies for the prevention of the spread of *C. auris*.

### 5.1. Surveillance

Public health surveillance has been a consistent strategy to reduce the transmission of pathogens. In the United States, *C. auris* clinical cases became designated as a nationally notifiable condition in 2018, with screening cases becoming notifiable in 2023 [45]. The CDC recommends reporting both clinical cases and surveillance (i.e., colonization) cases to optimize public health and infection prevention measures. Specifically for clinical cases, antifungal susceptibility testing is also recommended to monitor trends in resistance patterns [45,46].

Although surveillance is a critical strategy, it is limited by being retrospective in nature. There have been increased attempts to highlight the potential for more prospective and active surveillance. For example, the Maryland Multidrug Resistant Organism Prevention Collaborative performed a statewide cross-sectional point prevalence survey of patients on mechanical ventilation in both acute care hospitals and long-term care facilities. In total, 470 patients were screened for *C. auris* with positive testing in 31 (6.6%) patients. Of note, patients in long-term care facilities were more likely to be colonized with *C. auris* compared to acute care hospitals (RR, 1.97, 95% CI 0.99–3.92, *p* = 0.05) [47]. In another study, 47 point prevalence surveys were conducted at 18 unique facilities in Chicago, Illinois, and the authors identified that the highest prevalence of *C. auris* colonization was present at ventilator-capable skilled nursing facilities (median prevalence, 66%) and long-term acute care hospitals (median prevalence, 31%). Of note, colonization was infrequent at short-term acute care hospitals (median prevalence, 0%) and skilled nursing facilities without ventilator capability (median prevalence, 2%) [48].

### 5.2. Screening

Screening has been identified as a key strategy to prevent the spread of cases and outbreaks. Screening practices can be implemented in response to the identification of new cases or more broadly as a prevention approach. To perform screening, the CDC recommends a compositive swab of a person’s bilateral axillae and groin [49]. As mentioned earlier, individuals can be asymptomatic and colonized with *C. auris*, which makes the establishment of screening practices particularly important. In fact, an international expert panel described screening as a component of best practice infection prevention strategy for *C. auris* [50].

Despite the recognition of the importance of *C. auris* screening in healthcare facilities, an Emerging Infections Network Survey revealed that only 37% of respondents conducted active screening [51]. The CDC has established guidance regarding screening, noting that patients or residents with an epidemiologic link to a case, patients with current or previous healthcare encounters, and patients with risk factors (i.e., mechanical ventilation, indwelling medical devices, receipt of complex medical care, frequent or lengthy stays in healthcare facilities, colonization by or infection with other multidrug-resistant organisms) for acquiring *C. auris* should be considered for screening [52]. However, more evidence of comprehensive screening programs is needed to demonstrate the importance and effectiveness of *C. auris* screening in healthcare facilities. To further evaluate the impact of *C. auris* screening, Rosa et al. described the implementation of in-house *C. auris* screening across a large integrated health system utilizing a pre- and post-intervention study design. In the pre-intervention group, patients were screened by a two-step process. Initially, a nursing questionnaire was utilized and if a risk factor was identified, *C. auris* screening was performed through send out polymerase chain reaction (PCR) testing. In the post-intervention period, the same questionnaire and risk factor assessment were utilized, but PCR testing was conducted in-house. The investigators demonstrated that the median number of monthly tests increased from 14.5 (interquartile range, 7.5–26) to 205.5 (interquartile range, 181–234) from pre- to post-intervention periods (*p* < 0.0001), respectively. The authors also showed more than a doubling of rates of *C. auris* cases present on admission and a decrease in the incidence of *C. auris* hospital-onset fungemia over the course of the study [53]. There is need for the further investigation of screening programs, as the complexity of interactions between *C. auris* and other bacterial organisms that make up the skin microbiome may obscure certain results. Also, the specific PCR testing method utilized is critical due to variability in different strains and clades of *C. auris*.

### 5.3. Isolation Precautions

The CDC has published guidance for infection prevention measures around *C. auris*, including hand hygiene- and transmission-based precautions. In acute care facilities, contact precautions are recommended, while either contact or enhanced barrier precautions are recommended for nursing homes and skilled nursing facilities. In terms of the duration of precautions, the CDC recommends continuing the precautions throughout the entire duration of stay in healthcare facilities [54]. This area is ripe for future research to determine the exact length of time that a patient needs to remain on precautions. There is evidence suggesting that contact precautions for methicillin-resistant *Staphylococcus aureus* and vancomycin-resistant *Enterococcus* can be safely discontinued [55,56,57], but *C. auris* has unique features enabling its rapid spread, as discussed in this review, making the decision to discontinue precautions extremely difficult.

### 5.4. Environmental Disinfection

Environmental disinfection is a measure recommended as part of comprehensive infection prevention programs. The CDC has established products designated as List P effective for *C. auris* disinfection [54,58]. Of paramount importance, traditional healthcare cleaning products, such as quarternary ammonium products, are ineffective against *C. auris* [59,60].

As mentioned earlier, the persistence of *C. auris* is a striking characteristic that has been a well-described feature involved in *C. auris* environmental contamination. Studies have described *C. auris* surviving on both wet and dry surfaces for 7–14 days and even being cultured from bedding [61,62,63,64].

In terms of addressing the problem of environmental contamination, there is great difficulty in determining the best agents and products for *C. auris* disinfection. Studies and experiments use different techniques, limiting comparisons and meaning that results cannot be translated to clinical scenarios in the real world. Ultimately, international recommendations have focused on use of sporicidal agents, chlorine-based disinfectants, and hydrogen peroxide vapor for daily and terminal cleaning [64]. Additional strategies are urgently needed.

Ultraviolet light is one such strategy emerging with mixed results. In one study, a wall-mounted far ultraviolet C light technology reduced aerosolized bacteriophage and vegetative bacterial pathogens with limited efficacy against *C. auris* [65]. In another study, a quantitative testing method was used to evaluate the efficacy of ultraviolet C light on *C. auris* disinfection. Hard, nonporous surfaces were tested with glass slides utilized as carriers. The glass slides were inoculated with specific concentrations of *C. auris*. One set of carriers was exposed to the ultraviolet C light and the other set served as a control. The results demonstrated that a mobile ultraviolet C light tower achieved nearly 100% inactivation of *C. auris* within 7 min of continuous exposure. The authors commented that the ultraviolet C light system utilized was unique with its set of output bulbs and design-optimized reflectors, which enabled maximum exposure in the simulated environment [66].

### 5.5. Decolonization

Decolonization for multidrug-resistant organisms is a strategy that has been studied and implemented. As an example, a cluster-randomized trial that included 28 nursing homes assessed universal decolonization versus routine bathing. The decolonization group consisted of chlorhexidine bathing and nasal povidone–iodine administration. The results demonstrated a significantly lower risk of hospital transfer due to infection for the decolonization group compared to the routine care group, with 9.7 patients needing to be treated to prevent one infection-related hospitalization [67]. In another study, a cluster-randomized crossover trial was performed in five ICUs at a single center to assess the effectiveness of daily chlorhexidine bathing. The study evaluated a primary composite outcome of incidence of healthcare-associated infections, and the results showed no difference for daily chlorhexidine bathing versus a control group [68].

For *C. auris*, there is no clear evidence for the ability to decolonize individuals, but there has been strong interest in potential decolonization strategies. If successful, decolonization would be an effective strategy to prevent spread from patients colonized with *C. auris*. Wu et al. evaluated several synthetic and natural antiseptics for potential efficacy against *C. auris*. The study found that chlorhexidine, nystatin, and povidone–iodine were fungicidal, with the natural products tea tree oil and manuka oil being fungicidal as well [69]. Clinically, in a small study, a chlorhexidine decolonization protocol was implemented. The protocol involved the daily bathing of case patients with 4% chlorhexidine gluconate and daily cleaning with a 4% chlorhexidine wipe for one week. In total, 38 cases were included in the study, and the results showed that decolonization was not successful for 76.3% of the cohort [70]. As a reason for the ineffectiveness of chlorhexidine protocols, some have proposed that decreased susceptibility to chlorhexidine may be related to protective interactions with other members of the skin microbiome [71]. In another clinical study, Rosa and colleagues implemented a modified bathing protocol for patients with *C. auris* as part of a quality improvement initiative. Isotonic hypochlorite solution was added to a bathing protocol for 24 patients included in the study. After 4 weeks, 81.2% patients remained colonized with *C. auris*, with 3 patients developing a localized rash [72]. In terms of ongoing questions, there may be risk related to the complete eradication of *C. auris* from the skin, as this may upset the balance within the microbiota.

## 6. Future Directions

Due to the well-described threat of *C. auris*, future research and guidance to prevent spread of *C. auris* are sorely needed. From a clinical perspective, additional evidence and implementation strategies for surveillance and screening are key areas for further work with gaps in knowledge currently present. From a research perspective, translational science to develop more effective strategies for *C. auris* disinfection and decolonization would be optimal.

Looking towards the future, wastewater surveillance is one area that may hold promise as a potential public health strategy and tool against *C. auris*. Wastewater surveillance was proven to be an innovative and effective surveillance strategy during the COVID-19 pandemic, serving as an early signal of increasing cases for communities [73,74]. Now, there is interest in the use of this tool for other emerging pathogens, such as *C. auris*. For example, in a large *C. auris* outbreak in Southern Nevada, investigators studied the applicability of wastewater surveillance. The investigators demonstrated that *C. auris* was present in 79% (72/91) of the wastewater samples analyzed with a significant difference in sample positivity between sewer sheds with *C. auris*-positive healthcare facilities and those without *C. auris*-positive healthcare facilities (*p* = 0.04) [75]. The application of newer technologies and tools to complement traditional public health measures is of great interest for the future direction of this field.

Finally, *C. auris* is a part of a global interest in the further study of non-albicans *Candida* spp. Specifically, the emergence of a greater amount of infections due to non-albicans *Candida* spp. creates challenges for clinicians due to the identification of increased azole resistance in many of these species [76]. ECMM will investigate non-albicans *Candida* spp. through use of a multicenter study including hospitals from all over the world. The study will focus on antifungal resistance and tolerance, along with their effects on patient outcomes [77]. Collaborative efforts such as these will shed new light on *C. auris* and inform public health practitioners and clinicians moving forward.

## 7. Conclusions

*C. auris* is an alarming global public health threat due to its ability to promote outbreaks and carry significant resistance to traditional antifungal agents. Public health strategies and tools are a significant part of the armamentarium involved in fighting this pathogen. Further work on identifying optimal surveillance and screening practices is critical, while the continued study of potential tools for disinfection and decolonization is urgently needed. Finally, the application of newer technologies to assist traditional public health measures in fighting *C. auris* is welcome going forward.

## Data Availability

This paper is a review article, and all references used for this paper are included.
